# T Cell Mediated Antibody lnvariance in an Immune Response Against A Bacterial Carbohydrate Antigen Requires CD28/B7–1 Costimulation

**DOI:** 10.1155/2001/87168

**Published:** 2001

**Authors:** André Rademaekers, Eckehart Kölsch, Christoph Specht

**Affiliations:** Institute for Immunology University of Münster Domagkstraβe 3A Münster D-48129 Germany

**Keywords:** α(1→3) dextran, CD28, antibody invariance, T/B cell interaction, idiotype, bacterial antigens

## Abstract

The humoral immune response against α(1→3) dextran (Dex) in BALB/c mice is characterized by the formation of predominantly IgM antibodies bearing the J558 idiotype. IgG antibodies do not appear in euthymic mice. In athymic animals however, the response proceeds to a vigorous IgG production. In euthymic mice formation of IgG is suppressed by J558 idiotype- specific regulatory T cells recognizing in association with I-E^d^ and in cognate T/B interaction the VH CDR3 derived peptide of the J558 idiotpye. Only B-2 lymphocytes produce IgG whereas B-1 cells do not participate in the production of this Ig class. Using a novel synthetic all α(1→3)-D-gluco configurated tetrasaccharide the Dex-specific B cells can for the first time be analyzed in FACS. In experiments using this newly designed low molecular Dex no signs of B cell apoptosis can be found. This demonstrates a true silencing of persisting Bγ memory cells and supports previous by adoptive transfer experiments. In this suppression an involvement of CD28/B7–1 interaction can be demonstrated which is a necessary costimulatory suppression signal in addition to the cognate TCR/peptide-I-E^d^ interaction between J558 Id-specific T cells and J558 idiotype beating B cells. This results in an activation of 178–4 Ts cells, leading to an overall suppression of the Dex-specific IgG isotype production on the one hand and on the other hand provides a signal for the survival and clonal expansion of J558 Id-positive B cells.


*Developmental Immunology*, 2001, Vol. 8(3-4), pp. 243-257
Reprints available directly from the publisher
Photocopying permitted by license only

© 2001 OPA (Overseas Publishers Association)
N.V. Published by license under
the Harwood Academic Publishers imprint,
par of The Gordon and Breach Publishing Group,
a member of the Taylor and Francis Group

### T Cell Mediated Antibody Invariance in an Immune Response Against A Bacterial Carbohydrate Antigen Requires CD28/B7–1 Costimulation

ANDRÉ RADEMAEKERS*, ECKEHART KÖLSCH and CHRISTOPH SPECHT

*Institute for Immunology, University of Münster, Domagkstraße 3A, D-48129 Münster, Germany*

The humoral immune response against α(1→3) dextran (Dex) in BALB/c mice is characterized by the formation of predominantly IgM antibodies bearing the J558 idiotype. IgG antibodies do not appear in euthymic mice. In athymic animals however, the response proceeds to a vigorous IgG production. In euthymic mice formation of IgG is suppressed by J558 idiotype-specific regulatory T cells recognizing in association with I-E$^d$ and in cognate T/B interaction the VH CDR3 derived peptide of the J558 idiotpye. Only B-2 lymphocytes produce IgG whereas B-1 cells do not participate in the production of this Ig class. Using a novel synthetic all α(1→3)-D-gluco configurated tetrasaccharide the Dex-specific B cells can for the first time be analyzed in FACS. In experiments using this newly designed low molecular Dex no signs of B cell apoptosis can be found. This demonstrates a true silencing of persisting Bγ memory cells and supports previous by adoptive transfer experiments. In this suppression an involvement of CD28/B7–1 interaction can be demonstrated which is a necessary costimulatory suppression signal in addition to the cognate TCR/peptide-I-E$^d$ interaction between J558 Id-specific T cells and J558 idiotype bearing B cells. This results in an activation of 178–4 Ts cells, leading to an overall suppression of the Dex-specific IgG isotype production on the one hand and on the other hand provides a signal for the survival and clonal expansion of J558 Id-positive B cells.

*Keywords:* α(1→3) dextran, CD28, antibody invariance, T/B cell interaction, idiotype, bacterial antigens

*Abbreviations:* Dex, α(1→3) dextran, $Dex_4$, [α(1→3)D-Glc]4-O/VVVFITC, Id, idiotype

### INTRODUCTION

The antigenic determinantes of a number of biologically important substances consist of carbohydrates. In the case of encapsulated bacteria the polysaccharide capsule is important for both the pathogenicity and immunogenicity of these organisms, enabling them to resist opsonization and clearance from the blood. Only when antibodies to the specific capsular polysaccarides are present, a complement mediated lysis of invading bacteria is possible. The immune response against these antigens carries a number of features which differ from those against protein antigens (Mosier and Subbarao, 1982; Mond et al., 1995). In detail the Dex-specific antibody response in euthymic BALB/c mice is largely restricted to IgM

* Address correspondence and proofs send to: Dr. Andrė Rademaekers, University of Münster, Institute for Immunology, Domagkstr. 3A, D-48129 Münster, Germany. telephone ++49-(0)251 83 5 62 82, telefax ++49-(0)251 83 5 62 85, e-mail rademae@uni-muenster.de antibodies carrying the J558 Id (Schuler et al., 1982, 1984). Athymic BALB/c nu/nu mice proceed instead to a vigorous IgG response equally dominated by the J558 Id. Further analysis show that Dex-specific B cells committed to the formation of IgG antibodies (Bγ memory cells) develop in euthymic mice but are suppressed *in situ* by specific T cells. This fact limits the humoral response to the sole production of IgM antibodies. For a generalization of the mechanisms of anti-polysaccharide responses it is important to understand how the isotype restriction in the Dex-specific J558 Id-dominated antibody response in BALB/c mice works. J558 Id-specific T cells control the isotype expression in this humoral response. They silence in a specific T/B cell interaction the primed Dex-specific B lymphocytes and suppress an IgG response (Clemens et al., 1998).

A J558 Id-specific T cell clone, 178–4 Ts, with the properties of T suppressor cells operating *in situ* has previously been isolated and its TCR been sequenced (Stäb et al., 1990; Austrup et al., 1993). Cells of this clone restrict the Dex-specific response to IgM in BALB/c nu/nu mice after adoptive transfer of and T cell reconstitution with 178–4 Ts cells. At the same time they cause an accumulation of anergized Dex-specific Bγ memory cells. Thus the T cell clone 178–4 Ts is a true representative of the regulatory T cells operating *in situ* in euthymic BALB/c mice (Schuler et al., 1984; Stäb et al., 1990). An investigation of the degree of heterogeneity of the TCR usage revealed that all Dex-primed BALB/c mice use for a putative controlling element a very similar, if not identical TCR as the one found in 178–4 Ts cells (Rademaekers and Kölsch, 1995). This suggests a tight, possibly germline encoded interaction between the J558 Id bearing Dex-specific B cells and their J558 Id-specific 178–4 Ts analogous T cell counterparts controlling the Dex-specific response.

In this study we evaluate parts of the mechanism of this interaction. On the one side the tight idiotypic connection between T and B cells silences in a cognate interaction isotype switched Bγ memory cells. On the other side it forms a gate preventing the appearence of sequence variability in the rare Dex-specific IgG antibodies which otherwise exists in the IgM pool (Clemens et al., 1998). Furthermore it brings into play a CD28/B7–1 interaction as source of a costimulatory signal in the cognate interaction between J558-specific 178–4 Ts analogous T cells and J558 idiotype bearing Dex-specific B cells (Specht et al., 1999) which results in a T cell mediated dominance of a sole idiotype leading to invariance of antibody diversity. This is in contrast to the response against protein antigens where diversification is assumed to be the basis for successful adoptive immune responses leading to affinity maturation within the antibody repertoire. In the case of bacterial antigens an increase of antibody diversity however may be of disadvantage and can result in a loss of protection (Benedict and Kearney, 1999).

### RESULTS

### The $V_H$ CDR3 region contains the T cell epitope required for cognate suppression

To investigate the requirements for activation of 178–4 Ts cells, synthetic peptides of various CDR3 sequences found in the Dex-specific IgG hybridomas (Clemens et al., 1998) were synthesized as 13- or 18mers (Table I). The 13 mers comprise the amino acids from position 91 to 103 of the VH region. The 18mers start at position 84. It should be noted that all peptides include amino acids which are known to be preferred amino acids or are anchor sequences for binding by I-$E^d$ (Rammensee et al., 1995). Immortalized XS52 dendritic cells of BALB/c origin as APC (Xu et al., 1995) were pulsed with these peptides and used for stimulation of 178–4 Ts cells. Only the two peptides containing the J558 CDR3 region are able to stimulate 178–4 Ts cells to produce IFN-γ (Figure 1). All other peptides, despite containing Ab sequence-derived naturally occurring alterations of the J558 CDR3 region, fail to stimulate IFN-γ production by 178–4 Ts cells.

A sequence of great homology to the J558 CDR3 region from BALB/c mice is located in the D/$J_H$ junction region (amino acids 100–107) of the α(1→6)

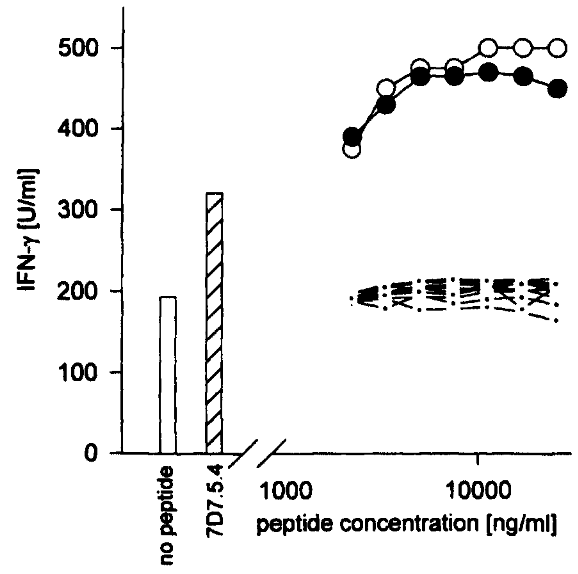

FIGURE 1 Importance of the J558 $V_H$ CDR3 region in the 178–4 Ts regulatory response. A confluent layer of immortalized XS52 dendritic cells as APC was fed with various amounts of different synthetic 13 or 18mer peptides respectively (see Table I). 178–4 Ts cells (2 × $10^4$) were added. After 48 h the activation of 178–4 Ts cells were measured by monitoring supernatants for IFN-γ. Only the 13mer (○) and the 18 mer (•) J558 peptide stimulate 178–4 Ts cells, whereas all other peptides fail to do so (lower lines). Controls are dendritic cells without antigen (open bars) or cultured with 10 μg/ml purified 7D7.5.4 mAb dextran B512F-specific IgM hybridoma F6H3 from a C57BL/6 mouse (Fernandez, 1994). Table I shows 2 synthetic peptides comprising this region in the 8 C-terminal aminoacids of the peptide. They do not stimulate 178–4 Ts cells (Figure 1). It should be noted that neither immunization with dextran B1355S nor with dextran B512F can expand 178–4 Ts analogous T cells in C57BL/6 mice (Clemens et al., 1998).

### Cognate T and B cell interaction in the suppression of the Dex-specific IgG response involves costimulation by CD28

Previous studies have shown that the interaction between J558-specific 178–4 Ts analogous T cells and J558 Id-bearing Dex-specific B cells prevents the appearance of IgG antibodies. In this interaction the J558 Id $V_H$ CDR3 region is recognized in association with I-$E^d$ (Austrup et al., 1993; Clemens et al., 1998). The use of knock out mice shows in addition a participation of CD28 membrane molecules in this suppression. BALB/c CD28$^{-/-}$ mice lacking the gene for CD28 mount an IgG response comparable to the one of BALB/c nu/nu mice (Figure 2a). Inhibition of CD28/B7–1 interaction, by the appropriate mAb IG10 also reduces suppression (Figure 2b). This experiment also includes immunization of BALB/c IFN-γ$^{-/-}$ animals lacking the gene for IFN-γ. Their response is equal to the one of BALB/c mice confirming previous experiments demonstrating that IFN-γ is not the mediator of T cell mediated suppression in the Dex-specific response (Clemens et al., 1998).

### IgG isotype suppression in the Dex-specific response is mediated by 178–4 Ts cells both in vivo and in vitro

Addition of 178–4 Ts cells to cultures of purified B cells from different mouse strains (Figure 3a), or reconstitution of BALB/c nu/nu mice with these regulatory T cells (Figure 3b) leads to a strong suppression of plasma cells producing the IgG isotype. These data demonstrate that 178–4 Ts cells regulate the isotype expression in the immune response against Dex very effective both *in vivo* and *in vitro*. The silenced B cells can be activated again by adotive transfer experiments (Schuler et al., 1982) or by drug administration (Austrup et al., 1991).

### Localisation of the Dex-specific B cells programmed for IgG production

Both B-1 (Förster and Rajewski, 1987) and B-2 (Schuler et al., 1984) lymphocytes can produce Dex-specific antibodies. In order to analyze the origin of the IgG producing cells peritoneal exudate and spleen cells were sorted according to their expression of CD5 and B220 molecules. Figure 4 shows that both B-1 and B-2 cells produce Dex-specific IgM. The Dex-specific IgG producers are only found in the B-2 population. In accordance with this assignment is

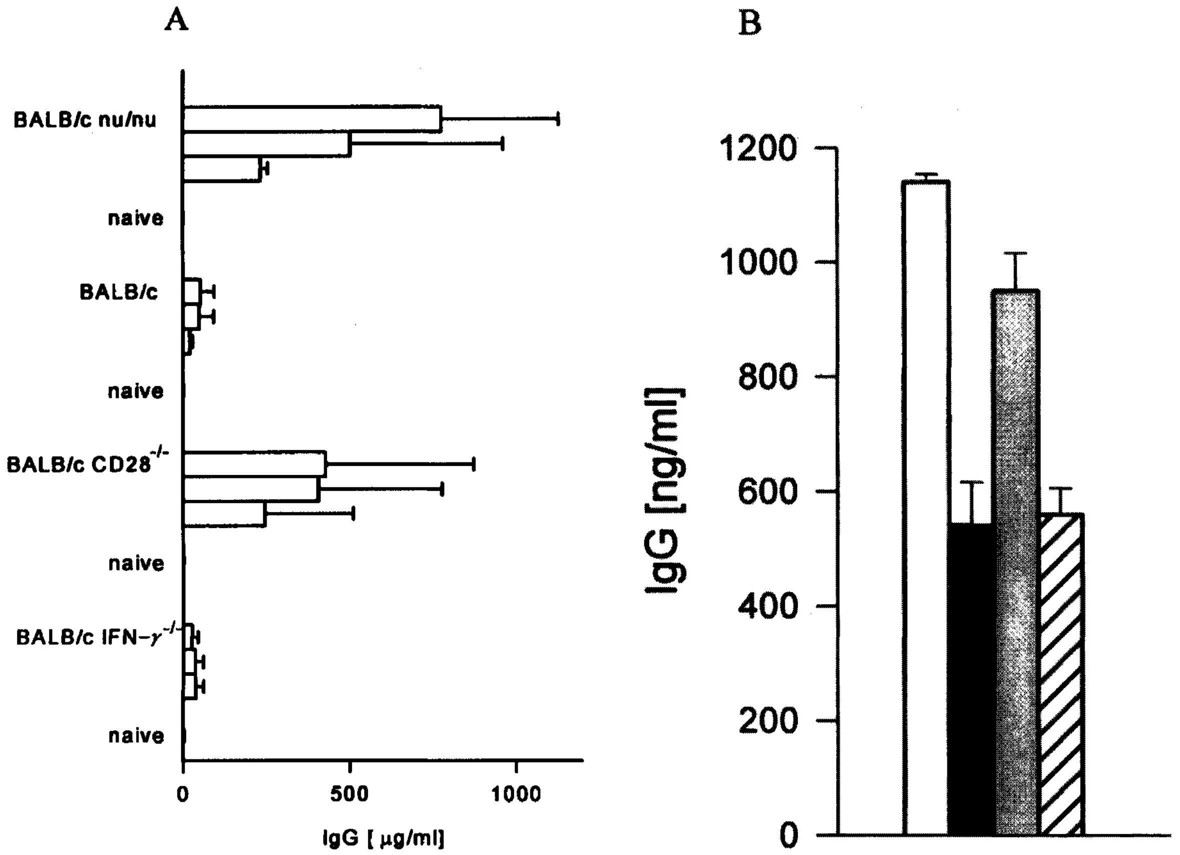

FIGURE 2 A: CD28$^{-/-}$ mice produce Dex-specific IgG comparable to BALB/c nu/nu mice and IFN-γ does not participate in the suppression of the Dex-specific IgG response. Mice were immunized three times biweekly with Dex. Sera were taken 12 days after each immunization. Bars represent means and SEM of the values from 5 mice. B: Presence of anti B7–1 mAb in cocultures of 178–4 Ts and Dex-primed BALB/c nu/nu spleen cells reduces suppression. Open bar: Spleen cells from primed animals alone; black bar: spleen cells and 178–4 Ts cells; grey bar: coculture in presence of anti B7–1 mAb; dashed bar: coculture in presence of anti B7–2 mAb. Means and ±SEM of three culture experiments are given. Note that in (A) serum antibody titers, whereas in (B) antibody titers from culture supernatants are given the finding, that all Dex-specific IgG producing hybridomas derived from euthymic as well from nude mice, and previously analyzed with regard to their molecular heterogeneity (Clemens et al., 1998) belong to the latter group.

### Suppression of Dex-specific Bγ memory cells is not due to apoptosis

Using $Dex_4$ it is also possible to analyze the status of Dex-binding B cells. Dex-specific B cells from immunized animals show no sign of apoptosis. In the total splenic B cell population are 25.9 % apoptotic cells. Among the apoptotic cells only 3.6% are Dex-specific B cells in contrast to 15.1% in the total B cell population (Figure 5a). Thus 4 to 5 times less apoptotic cells are found in the Dex-specific population as compared to the total B cell population. Similarly less than 4% of apoptotic B-1 and B-2 cells are found using the cell death detection kit (Figure 5b).

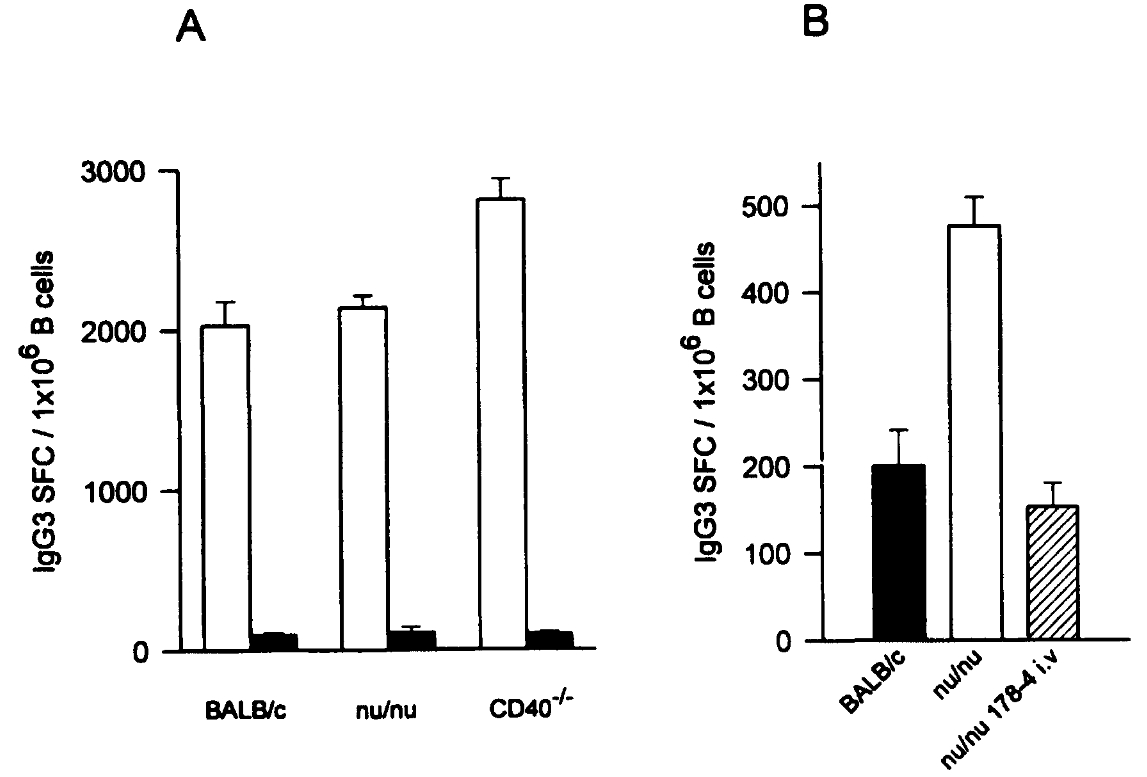

FIGURE 3 Spot-ELISA for anti Dex IgG3 producing plasma cells. A: Purified B cells from spleens of immunized BALB/c, BALB/c nu/nu and CD40$^{-/-}$ mice after six days of culture with (black bars) or without 178–4 Ts cells (white bars). B: B cells from Spleens of Dex-immunized euthymic BALB/c (black bar), athymic BALB/c nu/nu (white bar) or nu/nu mice reconstituted by intraperitoneal injection of $4 \times 10^7$ 178–4 Ts cells (hatched bar) *ex vivo* 12 days after immunization. Data represent the mean ± SEM of three independent experiments

### 178–4 Ts cells are necessary for survival and expansion of J558 Id bearing B cells

Previous data have shown that only B cells bearing the J558 Id are recognized by 178–4 Ts cells, whereas other Id are not perceptible for these T cells (Clemens et al., 1998). We now demonstrate an expansion of this Id mediated by 178–4 Ts cells. Highly purified B cells from Dex-immunized BALB/c mice, containing less than one percent T cells, were cultivated in the presence or absence of 178–4 Ts cells. Addition of 178–4 Ts cells to the B cell cultures does not only lead to a strong suppression of IgG3 production but also leads to a massive increase of the percentage of J558 Id positive B cells among IgG3 expressing cells. Upregulation of the J558 Id can be demonstrated *in vitro* where after 6 days of culture almost the whole IgG3 producing B cell population carries the J558 Id (Figure 6a). These B cells must be derived *in vitro* from the IgM pool, since depletion of IgG positive cells during the course of B cell purification has no influence neither on the IgG suppression nor the expansion of the J558 Id (Figure 6b). The development of J558 Id positive IgG producing B cells must be due to 178–4 Ts cells, because B cell culture in the absence of 178–4 Ts cells show a low percentage (30–40%) of J558 Id bearing B cells (Figure 6a). The same low percentage is found in athymic BALB/c nu/nu mice (Figure 7) where no regulatory T cells are present. Thus the presence of 178–4 Ts cells leads to a shift of the Id balance towards prevalence of the J558 idiotype. As shown in Figure 7 reconstitution of

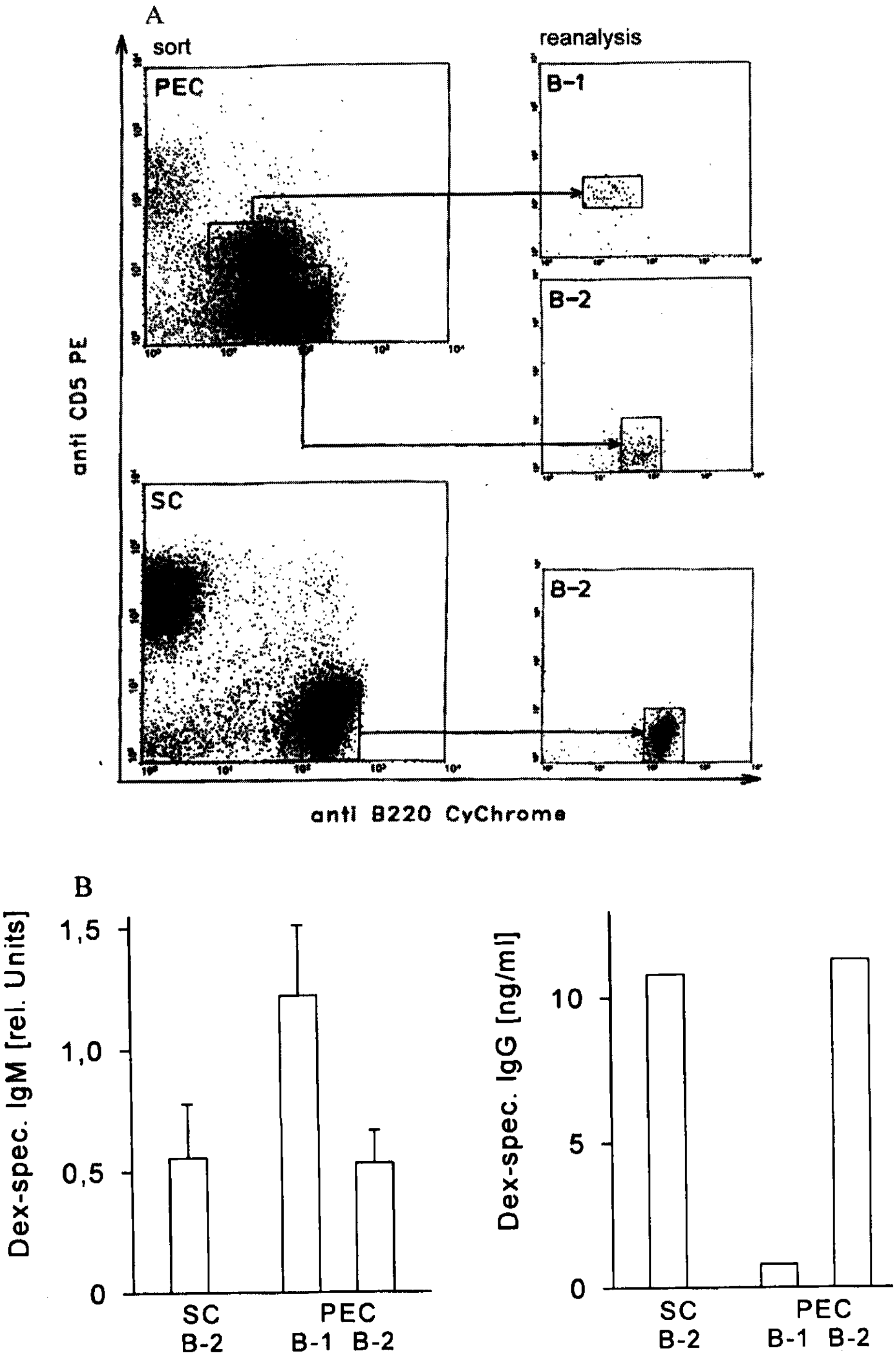

FIGURE 4 Only conventional B-2 lymphocytes from Dex-immunized BALB/c mice are the producers of Dex-specific IgG antibodies. (A) FACSort of PEC and SC into B-1 and B-2 cells. (B) Dex-specific IgM and IgG antibodies in the culture supernatants of the B cells sorted in A

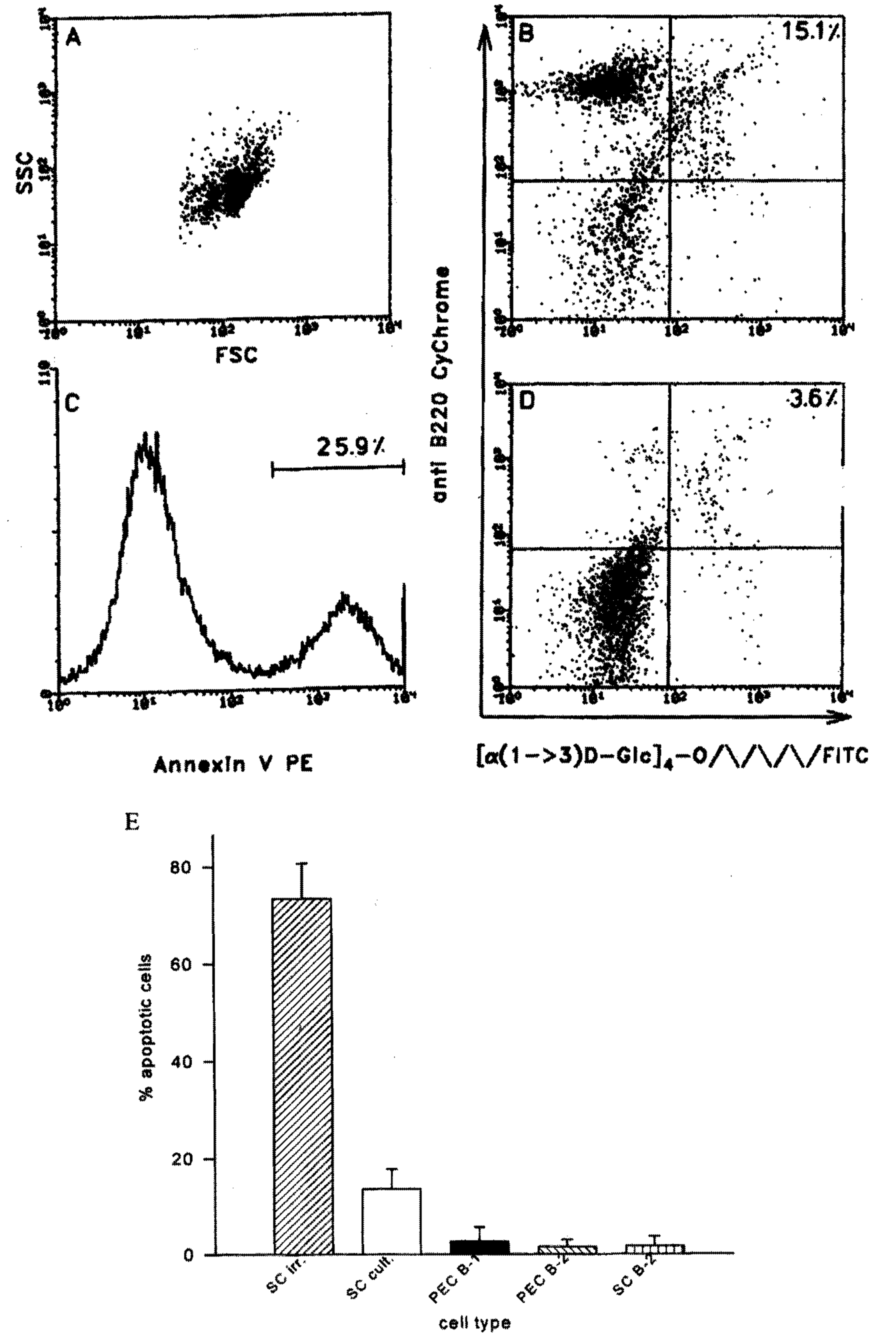

FIGURE 5 Determination of apoptotic cells in the spleen of Dex-immunized mice. A-D: Three colour staining of spleen cells of Dex immunized BALB/c mice. The total lymphocytes (A) were stained for B220 expression and Dex4 binding (B) as well as Annexin V binding (C). Annexin V positive cells were also analyzed for B220 expression and Dex4 binding (D). The percentage of apoptotic cells is lower in the Dex-specific population (D) than in the total spleen cell population (C). E: B-1 and B-2 cells from PEC and B-2 cells from spleen as well as irradiated (SC irr.) or cultured (SC cult.) spleen cells were probed for apoptosis using the in situ cell death detection kit. Means and ±SEM of 20 countings from two independent experiments were determined

BALB/c nu/nu mice with 178–4 Ts before immunization with Dex leads to a strong increase of J558 Id positive B cells, comparable to the situation in euthymic BALB/c mice. Simultaneously, B cells programmed for IgG production are reduced in these mice. Thus the regulatory T cells are responsible for the expansion of this major Id *in vivo* and *in vitro*. The large amount of J558 Id positive IgG3 producing B cells in BALB/c mice (Figure 7) is strongly reduced after 6 days of culture in the absence of 178–4 Ts cells (Figure 6). In contrast the percentage of J558 Id positive B cells remains at this high level or is even slightly enhanced when 178–4 Ts cells were added to the culture. The rate of IgG suppression as measured by spot-ELISA (Figure 3) is evidently higher than measured by *in situ* hybridization (Fig. 6 and 7). The number of Dex-specific plasma cells measured by spot-ELISA is for several reasons not comparable to the amount of all IgG3 mRNA expressing B cells detected by *in situ* hybridization. We used the *in situ* hybridization technique for the analysis of the major J558 Id since this analysis is the only way to discriminate the J558 Id positive B cells unequivocaly from cells with related antigen receptors.

### DISCUSSION

Bacterial polysaccharides like dextrans belong to a group of antigens which have been classified as "thymus-independent" (TI) antigens. The immune response against these widespread distributed antigens carries an number of features which differ from those against protein antigens (Mosier and Subbarao, 1982). Despite of being grouped to the thymus independent antigens the immune response against Dex is controlled by idiotype specific T cells and largely confined to the sole production of Dex-specific IgM Ab. In contrast to this situation in euthymic BALB/c mice, athymic BALB/c nu/nu mice upon immunization do not only produce IgM but proceed to a vigorous IgG Ab response. A T cell clone which is a true representative of the regulatory T cells operating in situ by all criteria tested, has previously been established (Stäb et al., 1990; Austrup et al., 1993).

As shown in Figure 1 this clone, 178–4 Ts, recognizes the $V_H$ CDR3 region of the J558 idiotype. Only a peptide comprising the J558 Id sequence (Table I) is recognized by 178–4 Ts cells whereas peptides of all other idiotypes with alterations in the CDR3 region fail to stimulate 178–4 Ts cells. The previous notion that cognate interaction between J558 Id-specific T cells and J558 Id bearing Dex-specific B cells is supported by additional evidence: The use of two different types of knock out mice, one strain lacking CD28 and the other lacking IFN-γ, indicates that CD28 must provide a critical signal (Figure 2a). In the Dex-specific response it is obvious that the $CD28^{-/-}$ mice respond like BALB/c nu/nu mice. Since there is an evidence that CD28 sustains the late T cell proliferative response and is necessary for longterm survival of T cells (Sperling et al., 1996) it follows that the Dex primed $CD28^{-/-}$ mice finally behave like animals specifically depleted of 178–4 Ts analogous T cells. The involvement of CD28/B7–1 interaction (Figure 2b) suggests in addition that a true costimulatory suppression signal is required for maintaining the silencing of B cells (Specht et al., 1999). In contrast purified B cells from $CD40^{-/-}$ mice behave like normal BALB/c mice indicating that there is no requirement for a CD40 costimulatory signal (Figure 3a). The low IgG titer in IFN-$\gamma^{-/-}$ as compared to normal mice (Figure 2a) is not surprising since previous experiments had already excluded IFN-γ as a mediator of suppression in the anti-Dex response (Clemens et al., 1998). The immune response against Dex is restricted to the sole production of IgM Ab in euthymic BALB/c mice whereas athymic BALB/c nu/nu mice proceed to a vigorous IgG response (Schuler et al., 1982, 1984). This situation can be simulated in cultures of purified B cells (Figure 3a). Reconstitution of BALB/c nude mice with 178–4 Ts cells before immunization leads to a massive suppression of the Dex-specific IgG response comparable to the level of euthymic BALB/c mice (Figure 3b). Addition of 178–4 Ts cells to cultures of purified B cells of both BALB/c and BALB/c nude mice leads to a strong suppression of IgG producing plasma cells. In this phase of silencing steps of differentiation must occur which result in the formation of Bγ memory

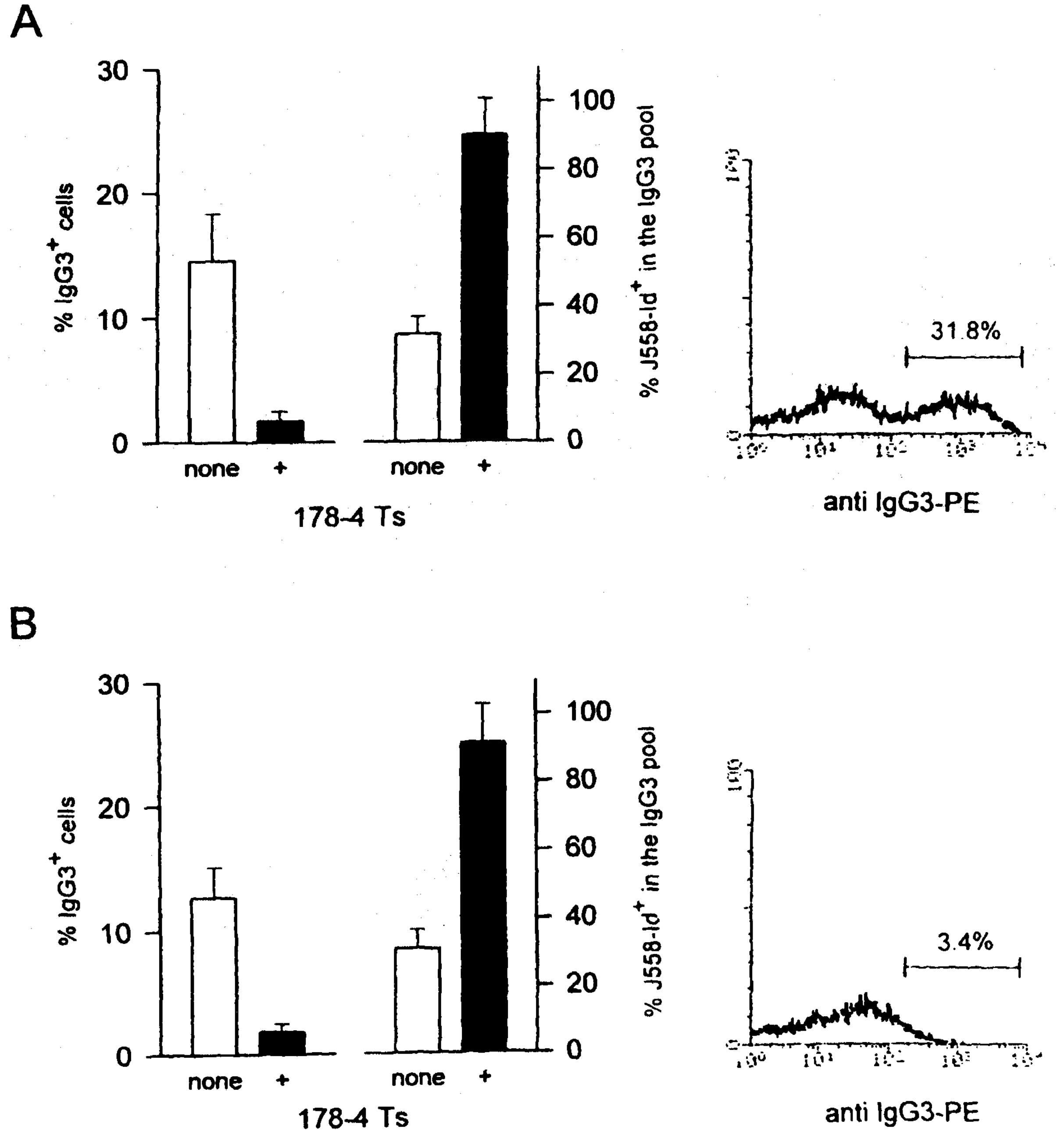

FIGURE 6 *In situ* analysis of cultivated B cells. A: Spleen cells from Dex-primed BALB/c mice were purified by MACS up to 96% and cultured for six days. 30% of these purified B cells are of the IgG isotype (A, right panel). In cultures with 178–4 Ts cells (black bars) IgG mRNA expression is impaired and the Id balance amoung the IgG pool is shifted to the sole expression of J558 Id (A, left panel). The IgG producing cells develop under control of 178–4 Ts cells *in vitro* from the IgM pool, since depletion of $IgG^+$ B cells before the culture (B, right panel) has no effect neither on IgG production nor on J558 expression (B, left panel). Data represent the mean ± SEM of three independent experiments cells. 178–4 Ts cells are assumed to be involved in this process. The experiments reported in this paper focus on "conventional" B-2 lymphocytes as the target of suppression. Although both B-1 and B-2 cells are capable to produce Dex-specific antibodies, only peritoneal and splenic B-2 cells produce IgG. Peritoneal B-1 cells are limited to IgM. The lack of Dex-specific IgG in B-1 cells (Figure 4b) strengthens the case that the T cell control is only operating at the level of B-2 cells, thus in the B cell compartment capable of affinity maturation in germinal centers during the course of immunization (Kelsoe, 1987). The use of a low molecular weight oligosaccharide ($Dex_4$) with three $\alpha(1\rightarrow3)$ glycosidic linkages in connection with a chromophore linked to it through a spacer allows now experiments at a cellular level. Using the new method it can be shown that among spleen cells of Dex-immunized mice virtually no Dex-specific B cells undergo apoptosis (Figure 5a). The low incidence of apoptotic cells is not unexpected since it is a characteristic of this immune response that Bγ memory cells accumulate in Dex-immunized mice remain silenced but can be activated upon adoptive transfer (Schuler et al., 1982) or by drug application *in situ* (Austrup et al., 1991).

As shown in Figure 6 addition of 178–4 Ts cells to the B cell cultures does not only lead to a strong suppression of the IgG production but also leads to a massive increase of the percentage of J558-idiotype positive B cells among IgG3 expressing cells (Figure 6). Thus the presence of 178–4 Ts cells leads to a shift of the Id balance towards the prevalence of the J558 Id. As shown in Figure 7 reconstitution of BALB/c nu/nu mice with 178–4 Ts cells before immunization with Dex leads to a strong increase of J558 Id positive Bγ cells comparable to the situation in euthymic BALB/c mice. Simultaneously B cells programmed for IgG production are reduced in these mice. Thus the regulatory T cells are responsible for the survival and expansion of B cells carrying this major idiotype *in vivo* and *in vitro*.

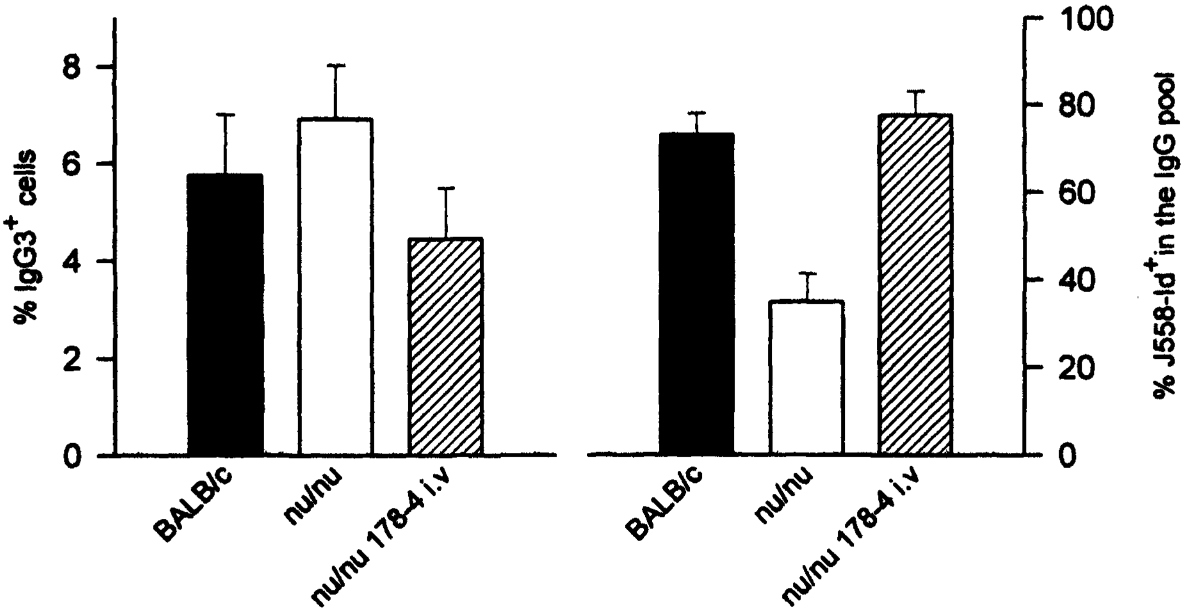

FIGURE 7 *In situ* hybridization of purified B cells. B cells from spleens of Dex-immunized euthymic BALB/c (black bars), athymic BALB/c nu/nu (white bars) or nu/nu mice reconstituted by intraperitoneal injection of 178–4 Ts cells (hatched bars) were monitored *ex vivo* for IgG mRNA expression and J558 Id distribution by *in situ* hybridization. In mice with 178–4 Ts or analogous T cells, IgG production is reduced, whereas the Id distribution amoung these cells is altered towards the sole expression of the J558 Id. The results represent the data ± SEM of three independent experiments

TABLE I Synthetic peptides of different hybridoma $V_H$ CDR3 regions used for T cell stimulation experiments

| *Peptides*[a] | *Sequence* | | | | | | | | | | | | | | |
|---|---|---|---|---|---|---|---|---|---|---|---|---|---|---|---|
| J558 | | **Tyr** | Cys | Ala | **Arg** | **Asp** | Arg | Tyr | Trp | Tyr | Phe | **Asp** | Val | Trp | |
| | Ser Glu Asp Ser Ala Val Tyr | **Tyr** | Cys | Ala | **Arg** | **Asp** | Arg | Tyr | Trp | Tyr | Phe | **Asp** | | | |
| MOPC 104 E | | **Tyr** | Cys | Ala | **Arg** | **Asp** | Tyr | Asp | Trp | Tyr | Phe | **Asp** | Val | Trp | |
| | Ser Glu Asp Ser Ala Val Tyr | **Tyr** | Cys | Ala | **Arg** | **Asp** | Tyr | Asp | Trp | Tyr | Phe | **Asp** | | | |
| 2F8 | | **Tyr** | Cys | Ala | **Arg** | **Asp** | Arg | Phe | Trp | Tyr | Phe | **Asp** | Val | Trp | |
| F8 | | **Tyr** | Cys | Ala | **Arg** | **Asp** | Gly | Tyr | Trp | Tyr | Phe | **Asp** | Val | Trp | |
| 5A8 | | **Tyr** | Cys | Ala | **Arg** | **Asp** | Asp | Gly | Asn | Tyr | Phe | **Asp** | Tyr | Trp | |
| N11H6.1 | | **Tyr** | Cys | Ala | **Arg** | **Asp** | Tyr | Asp | Tyr | Asn | Phe | **Asp** | Tyr | Trp | |
| 7C6.1 | | **Tyr** | Cys | Ala | **Arg** | **Asp** | Asn | Asn | Tyr | His | Phe | **Asp** | Tyr | Trp | |
| "artiticial" sequence | | **Tyr** | Cys | Ala | **Arg** | **Asp** | Glu | Glu | Trp | Tyr | Phe | **Asp** | Val | Trp | |
| | Ser Glu Asp Ser Ala Val Tyr | **Tyr** | Cys | Ala | **Arg** | **Asp** | Glu | Glu | Trp | Tyr | Phe | **Asp** | | | |
| F6H3 | | | Tyr | Asp | Tyr | **Asp** | Gly | Tyr | Trp | Tyr | Phe | **Asp** | Val | Trp | Gly |
| | Ser Glu Asp Ser Ala Val | **Tyr** | Tyr | Asp | Tyr | **Asp** | Gly | Tyr | Trp | Tyr | Phe | **Asp** | Val | | |

a. Peptides (13 or 18mer) share amino acid positions 84-101 or 91-103 of the $V_H$ region of respective Dex-specific Ab. Anchor sequences or preferred amino acids for I-$E^d$ are boldly marked. The lower two peptides contain a sequence found in the C57BL/6 Dex B512F specific IgM hybridoma F6H3 (Fernandez, 1994).

We propose the following mechanisms of immune regulation of 178–4 Ts analogous T cells in the response against bacterial polysaccharides (Figure 8). J558-Id bearing B cells present a distinct J558 peptide which comprises an amino acid sequence of the CDR3 region in an I-$E^d$ restricted manner are recognized by 178–4 Ts cells. This T/B cell interaction stimulates the 178–4 Ts cells to upregulate the CD28 costimulatory surface molecule. A costimulation through CD28/B7–1 interaction leads to an activation of these regulatory T cells which results in an overall suppression of the Dex-specific IgG isotype production on the one hand and on the other hand delivers a signal for the survival and clonal expansion of J558 Id-positive B cells. The dominance of this major idiotype leaves only little room for the appearance of B cells carrying other idiotypes. A less diverse germline encoded antibody repertoire in immune responses against bacterial polysaccharides is necessary for the maintenance of integrity of the immune system. An increase of antibody variability may result in a loss of protection against bacteria.

The idiotype-specific T cell 178–4 Ts maintains the integrity of a protective immune response against carbohydrate antigens by limiting the antibody diversity. Thus T cell mediated antibody invariance ensures a rapid and effective response and is of advantage in the immune response against bacterial carbohydrate antigens. This is in contrast to protein antigens where diversification is assumed fo be the basis for succesful adoptive immune responses leading to affinity maturation within the antibody repertoire (Figure 9). In the case of bacterial antigens an increase of antibody diversity however can result in a loss of protection (Benedict and Kearney, 1999).

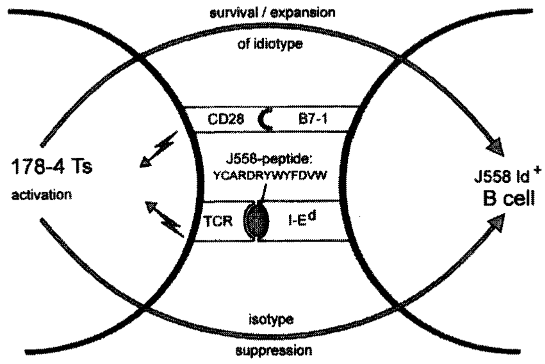

FIGURE 8 Postulated model for an Id-specific T/B cell interaction in the $\alpha(1\rightarrow3)$ Dex-specific response. 178–4 Ts cells specifically recognize an I-$E^d$ MHC II-restricted peptide derived from the heavy chain CDR3 region of the J558 Id on B cells. Costimulation through CD28/B7–1 interaction leads to activation of the T cells, which do not only deliver a signal for the suppression of the IgG isotype, but additionaly for the survival and expansion of J558 Id-bearing B cells

### MATERIALS AND METHODS

### Mice

BALB/c-AnNIcr and BALB/c-AnNIcr nu/nu mice were purchased from Charles River, Sulzfeld, Germany, or bred at the institute animal facilities. BALB.$Igh^b$ mice were derived from a breeding nucleus originally kept at the University of Konstanz (Germany). BALB/cJ, BALB/c CD28 <tmlMak> ($CD28^{-/-}$), BALB/CANNCR CD40 <TM1KIK> ($CD40^{-/-}$) and BALB/c Ifng <tmlTs> (IFN-$\gamma^{-/-}$) were purchased from the Jackson Laboratory, Bar Harbor, Maine (USA). Animals were used for experiments at an age of 8–12 weeks. In several cases BALB/c nu/nu mice were reconstituted with $4 \times 10^7$ 178–4 Ts cells by i.v. injection in the tail vein one day before immunization.

### Antigens and immunization

Dextran B1355S (Dex), average molecular weight $4 \times 10^7$ Dalton, from *Leuconostoc mesenteroides* containing 43% $\alpha(1\rightarrow3)$-D-glucopyranosyl linkages was used as antigen. It is a kind gift from Dr. M.E. Slodki (Northern Regional Research Laboratory U.S.D.A., Peoria, Ill. USA). Mice were usually immunized by a single i.p. injection of 10 μg Dex in saline. Spleen cells and peritoneal exudate cells (PEC) were taken 12 days after immunization. For the experiment shown in Figure 2 two additional immunizations were done at days 28 and 42 and in each case sera taken 12 days later. An all α(1→3)-D-gluco configurated tetrasaccharide was synthesized using anomeric thiiranium-intermediates as glycosyl donors. Fluorescein was linked by a spacer to the reduced end of the oligosaccharide. The compound is symbolized as $[\alpha(1\rightarrow3)D\text{-}Glc]_4$-O/VVVFITC ($Dex_4$). It was used in FACS analysis.

### Activation of J558 Id-specific T cells

Immortalized XS52 dendritic cells of BALB/c origin were cultured with 20% NS47 cell supernatants for one week on 96-well plates (Greiner, Nürtingen, Germany) to generate mature cells expressing MHC class II molecules (Xu et al., 1995). A confluent layer of XS52 as APC was incubated with various amounts of synthetic 13mer or 18mer peptides (Eurogentech, Brussels, Belgium) containing an anchor sequence or preferred aminoacids for $I\text{-}E^d$. Then $2 \times 10^4$ 178–4 Ts cells were added. After 48 h of cultivation the peptide-induced activation of 178–4 Ts cells was measured by monitoring supernatants for IFN-γ production.

### FACS analysis a) Sorting of B cells: Suspensions of hemolyzed spleen or peritoneal exudate cells of Dex-immunized BALB/c mice ($1 \times 10^7$ cells in 0,5 ml DMEM containing 10% FCS) were stained with 6 µl of FITC-labeled rat anti mouse B220 mAb RA3–6B2 (Pharmingen) and 4 µl of PE-labeled rat anti mouse CD5 mAb 53–7.3 (Pharmingen). Cell suspensions were sorted for B-1 and conventional B-2 B lymphocytes (Coulter Epics Elite). The FACSorter was calibrated using DNA-check beads (Coulter, Hialeah, Fl., USA.) Calibration points were set by eye. Coulter Elite software was used for sorting and analysis. Sorted B cells were cultured in DMEM containing 10% FCS, 10 µg/ml LPS (Sigma), 5 µg/ml rmIL-4 (Pharmingen) and 100 U/ml rmIL-5 (Pharmingen) on 24 well plates (Greiner). After 10 days of culture the supernatants were monitored for Dex-specific IgG and IgM by ELISA. For quantification of IgG, supernatants had to be pooled and were concentrated 10-fold using Millipore Ultrafree-MC-filters (30 000 NMWL) (Millipore, Eschborn, Germany) before ELISA detection. Freshly isolated B-1 and B-2 lymphocytes were also monitored for apoptosis.

b) FACScan of Dex-specific B cells: Hemolyzed suspensions of spleen cells from naive or Dex immunized mice ($2 \times 10^6$ cells in 300 µl BSS) were stained with 6 µl of $Dex_4$, 4 µl of CyChrome-labeled rat anti mouse B220 mAb RA3–6B2 (Pharmingen) and 4 µl of PE-labeled Annexin V (Southern Biotechnology Associates Inc., Birmingham, AL, USA). For detection of IgG-positive B cells, biotinylated anti-IgG3 R40–82 monoclonal Ab and Streptavidin-Phycoerythrine (Pharmingen) was used. Stainings of total cells were evaluated with Becton Dickinson FACScan, using LYSIS II software. All gates and regions were set by eye.

### Detection of apoptotic cells by fluorescence in situ hybridization

The detection of apoptotic cells was performed using the *In Situ* Cell Death Detection Kit Fluorescein from Boehringer Mannheim (Mannheim, Germany). Sorted spleen cells or peritoneal exudate cells from BALB/c mice immunized with Dex as well as 48 h cultivated spleen cells or irradiated spleen cells were centrifuged onto glass slides and fixed with 4% paraformaldehyde in PBS for 30 min. Cell permeabilization was achieved by incubating the slides in 0,1% Triton X-100, 0,1% sodium citrate for 2 min on ice. After washing with PBS a "TUNEL" reaction mixture containing terminal transferase to label free 3'OH-ends of genomic fragmented DNA with fluorescein – dUTP, was added to the samples. After incubating for 60 min at 37°C in the dark the slides were rinsed 3 × with PBS. Then the samples were directly analyzed by fluorescence microscopy and the number of apoptotic cells showing green fluorescence was counted independently by two persons. For each sample the mean ± SEM of counts from twenty areas was determined.

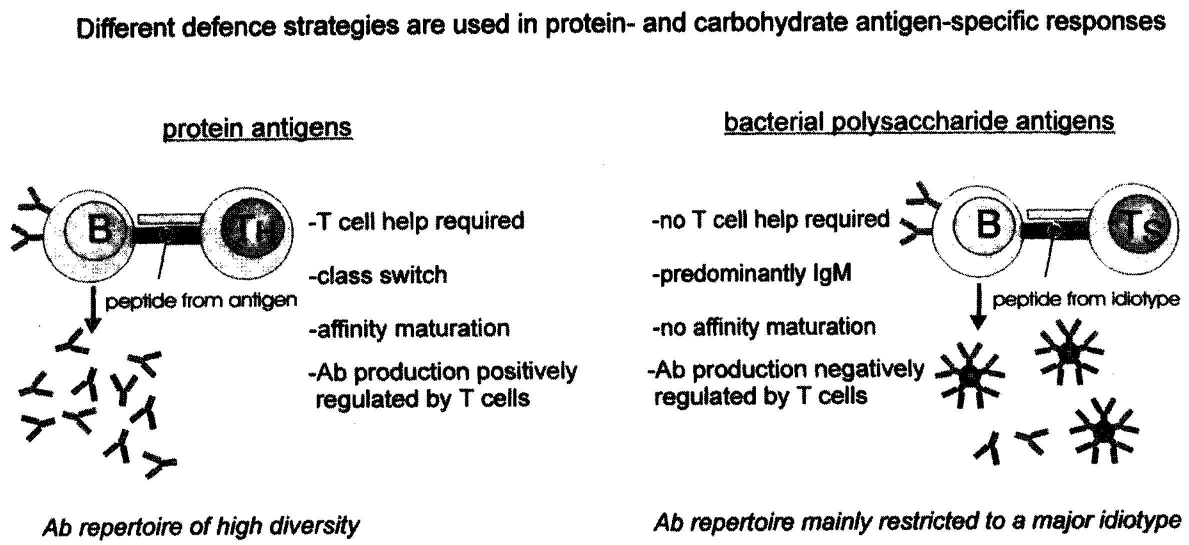

FIGURE 9 Summery of the relevant features discriminating immune responses of protein antigens from those against bacterial carbohydrates

**Purification of B cells and cell culture**

12 days after immunization with Dex, spleens were harvested and a single cell suspension was prepared. B cells were separated by MACS using an anti-mouse CD43 monoclonal Ab (Miltenyi Biotech, Bergisch Gladbach, Germany) (Gulley et al., 1988) alone or in addition with goat anti-mouse IgG monoclonal Ab (Miltenyi Biotech) (Simon et al., 1996). The T cell clone 178–4 Ts was maintained as previously described (Austrup et al., 1993). For some experiments $1 \times 10^6$ purified B cells from BALB/c or nude mice were cultivated for 6 days in the absence or presence of $3{,}3 \times 10^3$ 178-Ts cells. Cells were cultivated under standard conditions in DMEM containing 10% FCS. In some experiments B7–1 specific mAb 1G10 and B7–2 specific mAb P03 (Pharmingen, Hamburg, Germany) were used.

**Dex-specific spot-ELISA**

The majority of Dex-specific IgG Ab is of the IgG3 isotype, therefore we confine our analysis to this isotype although we know that other IgG isotypes are also equally affected. Determination of the frequency of IgG3 Ab-producing cells (SFC) among spleen cells *ex vivo* or after cultivation *in vitro* was performed using the spot-ELISA (Sedgwick and Holt, 1983). 96-well plates (Silent Screen, Nunc, Wiesbaden, Germany) were coated over night at 4 C with 60 μg/ml Dextran as Ag. $3 \times 10^5$ MACS-purified B cells *ex vivo* or from the 6 day culture were serially diluted and after incubation for 4 h tested for their production of Dex-specific IgG3. The biotinylated monoclonal Ab Biotin anti-mouse IgG3 (R40–82, Pharmingen, Hamburg, Germany) was used as secondary Ab. After addition of Streptavidin (ExtrAvidin alkaline phosphatase conjugate, Sigma, Deisenhofen, Germany) the Dex-specific SFC were detected by 5-Bromo-4-Chloro-3-Indoylphosphate/Nitroblue Tetrazolium staining.

**In situ hybridization**

In situ hybridization was performed on paraformaldehyde fixed cells as described before (Komminoth, 1992; Clemens et al., 1998). For the detection of IgG3 producing B cells a digoxigenin-marked oligonucleotide (5 -GATTCTCTTGATCAACTCA GTCTT-

GCTGGC-3) complementary to a sequence of the constant region was used and detected by anti-digoxigenin Ab (Boehringer Mannheim, Germany). A second oligonucleotide labeled with rhodamine (TRITC) at the 5 end (5 -CCGTGGTCCCTGCGCCCCA-GACA-3) complementary to the CDR3 region was used to identify J558 Id bearing B cells.

### *Acknowledgements*

This work was supported by the Deutsche Forschungsgemeinschaft through Sonder-forschungsbereich 310 (Teilprojekt C3).

### *References*

Austrup, F., Kucharzik, T. and Kölsch, E. (1991). Cremophor EL as an adjuvant affecting immunoglobulin class switch in the immune response to the thymus-independent antigen α(1→3) dextran B 1355 S. Immunology **73**:508–509.

Austrup, F., Kodelja, V., Kucharzik, T. and Kölsch, E. (1993). Characterization of idiotype-specific I-$E^d$-restricted T suppressor lymphocytes which confine immunoglobulin class expression to IgM in the anti-α(1→3) dextran B1355S response of BALB/c mice. Immunobiol. **187**:36–50.

Benedict, C.L. and Kearney, J.F. (1999). Increased junctional diversity in fetal B cells results in a loss of protective anti-phosphorylcholine antibodies in adult mice. Immunity **10**:607–617.

Clemens, A., Rademaekers, A., Specht, C. and Kölsch, E. (1998). The J558 $V_H$ CDR3 region contributes little to antibody avidity; however, it is the recognition element for cognate T cell control of the α(1→3) dextran-specific antibody response. International Immunol. **12**:1931–1942.

Fernandez, C. (1994). Identical $V_H$D and $DJ_H$ junctions in monoclonal antibodies derived in response to dextran B512 could be the result of developmental selection. Scand. J. Immunol. **40**:581–590.

Förster, I. and Rajewsky, K. (1987). Expansion and functional activity of Ly-$1^+$ B cells upon transfer of peritoneal cells into allotype-congenic, newborn mice. Eur. J. Immunol. **17**:521–528.

Gulley, M.L., Ogata, L.C., Thorson, J.A., Dailey, M.O. and Kemp, J.D. (1988). Identification of a murine Pan-T cell antigen which is also expressed during the terminal phases of B cell differentitation. J. Immunol. **140**:3751–3757.

Kelsoe, G. (1987). *In situ*studies of the germinal center reaction. Adv. Immunol. **60**:267–288.

Komminoth, P. (1992). Digoxigenin as an alternative probe labeling for *in situ* hybridization. Diagn. Mol. Pathol. **1**:142–150.

Mond, J.J., Lees, A. and Snapper, C.M. (1995). T cell-independent antigens type 2. Annu. Rev. Immunol. **13**:655–692.

Mosier, D.E. and Subbarao, B. (1982). Thymus-independent antigens: complexity of B-lymphocyte activation revealed. Immunol. Today 3:217–222.

Rademaekers, A. and Kölsch, E. (1995). Regulation of an anti-polysaccharide immune response in BALB/c mice through a tight T and B lymphocyte idiotypic connection. Eur. J. Immunol. **25**:623–626.

Rammensee, H.-G., Friede, T. and Stevanović, S. (1995). MHC ligands and peptide motifs: first listing. Immunogenetics. **41**:178–228.

Schuler, W., Lehle, G., Weiler, E. and Kölsch, E. (1982). Immune responses against the T-independent antigen α(1→3) dextran. I. Demonstration of an unexpected IgG response in athymic and germ-free-raised euthymic BALB/c mice. Eur. J. Immunol. **12**:120–125.

Schuler, W., Schuler, A. and Kölsch, E. (1984). Immune response against the T-independent antigen α(1→3) dextran. II. Occurence of B memory cells in the course of immunization with the native polysaccharide is T cell dependent. Eur. J. Immunol. **14**:578–585.

Sedgwick, J.D. and Holt, P.G. (1983). A solid-phase immunoenzymatic technique for the enumeration of specific antibody-secreting cells. J. Immunol. Methods **57**:301–309.

Simon, H.U., Yousefi, S., Domann-Scherrer, C.C., Zimmermann, D.R., Bauer, S., Barandun, J. and Blaser, K. (1996). Expansion of cytokine-producing CD4-CD8- T cells associated with abnormal fas expression and hypereosinophilia. J. Exp. Med. **183**:1071–1082.

Specht, C., Junker, R., Krüger, A., Rademaekers, A., Redlich, A. and Kölsch, E. (1999). Involvement of CD28 cosignaling in the T cell mediated suppression of the IgG antibody response against the TI-2 antigen α(1→3) dextran. Immunobiology **201**:49–63.

Sperling, A. I., Auger, J.A., Ehst, B.D., Rulifson, I.C., Thompson, C.B. and Bluestone, J.A. (1996). CD28/B7 interactions deliver a unique signal to naive T cells that regulates cell survival but not early proliferation. J. Immunol. **157**:3909–3917.

Stäb, F., Austrup, F. and Kölsch, E. (1990). Regulation of the anti-α(1→3) dextran IgG antibody response of BALB/c mice by idiotype-specific T suppressor lymphocytes. J. Immunol. **144**:53–59.

Xu, S., Ariizumi, K., Caceres-Dittmar, G., Edelbaum, D., Hashimoto, K., Bergstresser, P.R. and Takashima, A. (1995). Successive generation of antigen-presenting, dendritic cell lines from murine epidermis. J. Immunol. **154**:2697–2705.

